# 

**Published:** 1910-11

**Authors:** 


### CONTENTS.

ORIGINAL COMMUNICATIONS—

When Should an External Urethrotomy be Done. By T. M. McIntosh, Thomasville, Ga. .......... 381

Headaches and Neuralgias Due to Diseases of the Nose and Accessory Sinuses. By Hugh M. Lokey, M. D., Atlanta, Ga. .......... 387

Chronic Non-Suppurative Middle Ear Diseases. By Geo. H. Cooper, Opelika, Ala. 395

Some Variations in Appendicits. By Frank K. Boland, M. D., Atlanta, Ga. ...... 398

Vaginal Caesarian Section. Indications, Limitations and Technique. By R. R. Kime, M. D., Atlanta, Ga. .......... 403

Anterior Poliomyelitis. By Dr. Teo. Toepel, M. D., Atlanta, Ga. .......... 408

A Case of Pulmonary Tuerculosis Treated by Artificial Phemothorax. By Mary E. Lapham, M. D., Highland Camp Sanatorium, Highlands, N. C. ........ 413

COMMUNICATIONS .......... 416

EDITORIALS .......... 419

NEWS AND NOTES .......... 423

BOOK REVIEWS .......... 432

